# Use of a Modified Vector Model for Odor Intensity Prediction of Odorant Mixtures

**DOI:** 10.3390/s150305697

**Published:** 2015-03-09

**Authors:** Luchun Yan, Jiemin Liu, Di Fang

**Affiliations:** School of Chemistry and Biological Engineering, University of Science and Technology Beijing, Xueyuan Road 30, Haidian District, Beijing 100083, China; E-Mails: yanluchun@126.com (L.Y.); difang_happy@163.com (D.F.)

**Keywords:** air quality, aromatic compounds, odor intensity, odor interaction, sensory evaluation, vector model

## Abstract

Odor intensity (OI) indicates the perceived intensity of an odor by the human nose, and it is usually rated by specialized assessors. In order to avoid restrictions on assessor participation in OI evaluations, the Vector Model which calculates the OI of a mixture as the vector sum of its unmixed components’ odor intensities was modified. Based on a detected linear relation between the OI and the logarithm of odor activity value (OAV—a ratio between chemical concentration and odor threshold) of individual odorants, OI of the unmixed component was replaced with its corresponding logarithm of OAV. The interaction coefficient (cosα) which represented the degree of interaction between two constituents was also measured in a simplified way. Through a series of odor intensity matching tests for binary, ternary and quaternary odor mixtures, the modified Vector Model provided an effective way of relating the OI of an odor mixture with the lnOAV values of its constituents. Thus, OI of an odor mixture could be directly predicted by employing the modified Vector Model after usual quantitative analysis. Besides, it was considered that the modified Vector Model was applicable for odor mixtures which consisted of odorants with the same chemical functional groups and similar molecular structures.

## 1. Introduction

Volatile organic compounds (VOCs) are typical air pollutants in indoor/outdoor environments, and they usually account for a substantial proportion of total pollutant concentrations [1,2]. Most VOCs which have very low odor thresholds (the lowest concentration of an odorant that is perceivable by human nose) can easily cause pungent odors at low concentration levels [3,4]. Because of the interaction between these odorants, mixtures of many negligible odor pollutants will generate a stronger odor [5]. The odor pollution caused by these VOCs not only causes serious threats to human health, but it also lowers the quality of life. Thus, odor pollution has caused wide public concerns. Normally, chemical concentrations of some targeted compounds are measured through instrumental analysis methods. However, the overall air quality is easily overlooked [6], thus it is urgent to employ some more comprehensive and intuitive methods for air quality evaluation.

The sensory evaluation method has been widely employed in the assessment of odor pollution [7,8]. As one of the most important evaluation criteria, odor intensity (OI) judges the degree of odor pollution in a more quantitative way [9,10,11]. For instance, the intensities of odor are classified into eight or twelve levels in an odor intensity referencing scale (OIRS) in the American Society for Testing Material (ASTM) standards [12]. Usually, OI is rated by a panel of specialized assessors in an odor-free testing room. Accordingly, the measurement of OI is mainly performed as laboratory research because of its specific measuring condition requirements. In order to promote the application of OI evaluation, human assessors are supposed to be replaced and predictive OI methods have been widely explored. According to the literature, many OI prediction models have been proposed (e.g., Vector Model, U Model, Additivity Model, the Strongest Component Model, *etc.*) [13,14,15]. Laffort *et al.* performed a series of comparative experiments, and the Vector Model displayed better predictive performance and feasibility than other models [16]. The Vector Model was firstly proposed by Berglund *et al.* in 1973, and it suggests that the perceived intensity of mixtures equals the vector sum of the perceived intensity of their unmixed components [17]. The participation of human assessors is still necessary to use the Vector Model. Because of that, research on the relations between OI and the chemical concentrations of odorants has also been extensively reported. For example, Whelton *et al.* proposed a Weber-Fechner model between OI and log concentration of drinking water odorants; Liden *et al.* reported the relation between OI and concentration of pyridine by means of intermodal power functions [18,19]. However, the research on the relationships between OI and chemical concentrations is still mainly focused on individual odorants rather than odor mixtures.

In this paper, a series of laboratory tests have been performed to measure the OI and chemical concentrations of odor samples which contained both individual aromatic compounds and their mixtures. The experimental results derived from individual odorants were used to relate the perceptual measure (*i.e.*, OI) with the corresponding chemical concentration. Furthermore, the relation between OI of a binary odor mixture and the odor intensities of its unmixed components was investigated. Based on the above results, the Vector Model was modified by relating the OI of a mixture with the logarithm of odor activity value (lnOAV) values of its components. After measuring the chemical concentrations of some new odor samples, their corresponding OI values were predicted by employing the modified Vector Model, which thus provided a new method for OI evaluation that will probably help extend its application to more related fields.

## 2. Experimental Section

### 2.1. Odor Sample and Assessors

As a group of typical air pollutants found in indoor environments, the aromatic compounds were chosen as the target substances in this study. As listed in Table 1, benzene (B, 99.5%), toluene (T, 99.5%), ethylbenzene (E, 98.5%), *n*-propylbenzene (P, 98.5%), *o*-xylene (O, 98%), *m*-xylene (M, 98%) and styrene (S, 98%) were employed. All the stimuli were purchased from the J&K Scientific (Beijing, China). Each odorant was individually injected into a specialized plastic bag for odor tests (Sinodour, Tianjin, China), and used as a gas standard after the odorant had completely evaporated. Odor samples were prepared by transferring a certain amount of standard gas into a new plastic bag filled with purified air (bag volume = 3 L).

A sensory panel (nine assessors, five males and four females) were recruited from the University of Science and Technology Beijing. Their ages ranged from 21 to 29 years (mean = 25 years), and all of them had participated in several experiments using similar olfactory methods.

**Table 1:** List of odorants used for Vector Model modification.

Order	Odorant	Abbreviation	CAS#	ChemicalStructure	Odor Threshold/(mg/m^3^)
1	Benzene	B	71-43-2	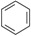	2.53
2	Toluene	T	108-88-3	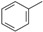	1.43
3	Ethylbenzene	E	100-41-4	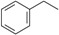	0.45
4	*n*-Propylbenzene	P	103-65-7	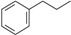	0.57
5	*o*-Xylene	O	95-47-6	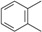	1.37
6	*m*-Xylene	M	108-38-3	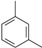	1.55
7	Styrene	S	100-42-5	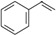	0.19

### 2.2. The Vector Model Methodology

The Vector Model can be seen as adjacent sides of a parallelogram where the lengths of the sides represent the perceived intensities of the unmixed components and the length of a diagonal through the figure represents the perceived intensity of the mixture [14]. Thus, the OI of a binary mixture is successfully related with the odor intensities of its unmixed constituents as the following equation [16]:

(1) $${\text{OΙ}}_{\text{ab}}^{2}={\text{OΙ}}_{a}^{2}+{\text{OΙ}}_{b}^{2}+2\times \text{cos }{\text{α}}_{\text{ab}}\times {\text{OΙ}}_{a}∙{\text{OΙ}}_{b}$$

where *a* and *b* indicates two different substances and OI_ab_ is the OI of their mixture. The interaction coefficient cosα (α is the angle between the two sides of a parallelogram) represents the degree of interaction between two unmixed components of a binary odor mixture. Normally, cosα is determined empirically for components of equal perceived intensities, and serves to predict the OI of remaining mixtures in a set. The corresponding equation for calculating cosα was as described below:

(2) $$\text{cos }{\text{α}}_{\text{ab}}=\frac{{\text{OΙ}}_{\text{ab}}^{2}-{\text{OΙ}}_{a}^{2}-{\text{OΙ}}_{b}^{2}}{2{\text{OΙ}}_{a}∙{\text{OΙ}}_{b}}$$

Usually, the cosα values differed among different odor mixtures. Besides, olfactory tests were still required to measure the cosα value before the corresponding application of the Vector Model. Thus, the Vector Model is barely used in actual OI prediction. For ternary and quaternary odor mixture, their corresponding Vector Models were as reported below [16]:

(3) $$\begin{array}{r} {\text{OΙ}}_{\text{abc}}^{2}={\text{OΙ}}_{a}^{2}+{\text{OΙ}}_{b}^{2}+{\text{OΙ}}_{c}^{2}+2\times \text{cos }{\text{α}}_{\text{ab}}\times {\text{OΙ}}_{a}∙{\text{OΙ}}_{b}\\ +2\times \text{cos }{\text{α}}_{\text{ac}}\times {\text{OΙ}}_{a}∙{\text{OΙ}}_{c}\\ +2\times \text{cos }{\text{α}}_{\text{bc}}\times {\text{OΙ}}_{b}∙{\text{OΙ}}_{c} \end{array}$$

(4) $$\begin{array}{r} {\text{OΙ}}_{\text{abcd}}^{2}={\text{OΙ}}_{a}^{2}+{\text{OΙ}}_{b}^{2}+{\text{OΙ}}_{c}^{2}+{\text{OΙ}}_{d}^{2}+2\times \text{cos }{\text{α}}_{\text{ab}}\times {\text{OΙ}}_{a}∙{\text{OΙ}}_{b}\\ +2\times \text{cos }{\text{α}}_{\text{ac}}\times {\text{OΙ}}_{a}∙{\text{OΙ}}_{c}\\ +2\times \text{cos }{\text{α}}_{\text{ad}}\times {\text{OΙ}}_{a}∙{\text{OΙ}}_{d}\\ +2\times \text{cos }{\text{α}}_{\text{bc}}\times {\text{OΙ}}_{b}∙{\text{OΙ}}_{c}\\ +2\times \text{cos }{\text{α}}_{\text{bd}}\times {\text{OΙ}}_{b}∙{\text{OΙ}}_{d}\\ +2\times \text{cos }{\text{α}}_{\text{cd}}\times {\text{OΙ}}_{c}∙{\text{OΙ}}_{d} \end{array}$$

As above mentioned, the OI of a mixture was only calculated as the vector sum of its unmixed components’ odor intensities, however, the OI values (e.g., OI_a_, OI_b_) in the Vector Model were not real vectors.

### 2.3. Experimental Procedure

**Figure 1:**
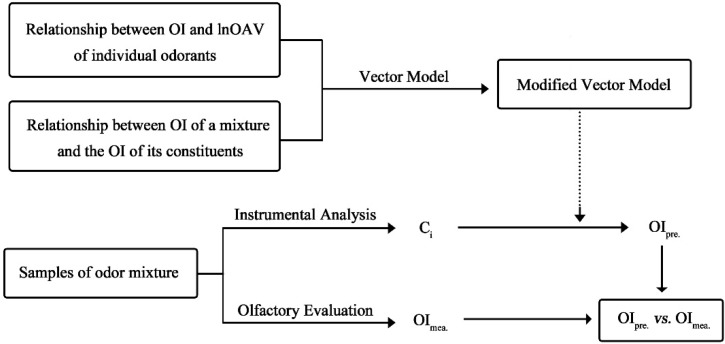
Experimental scheme of the Vector Model modification and its application in odor intensity prediction of odor mixtures.

The experimental scheme of this study is presented in Figure 1, and it can mainly be divided into two sections. In the first section, the relation between OI and lnOAV was firstly investigated by using a series of individual odor samples. The odor activity value (OAV) indirectly represented the concentration level of an odor sample and it was calculated as the ratio between the concentration of individual substance in a sample and the threshold concentration of this substance (*i.e.*, OAV = C_i_/C_Thr._). Based on that, the OI of an unmixed component in the Vector Model (e.g., OI_a_, OI_b_ in Equation (1)) was replaced with its corresponding lnOAV value. Simultaneously, the relationship between the OI of a binary odor mixture and the odor intensities of its constituents was explored. Based on the obtained results, the determination method of cosα was suitably simplified. Then, a modified Vector Model was established. In the second section, several odor mixtures were prepared in the form of a series of different odor samples. The OI of each odor sample was rated by a sensory panel (OI_mea._) and chemical concentrations of its constituents were also measured by gas chromatography. Through plugging the measured chemical concentrations in the modified Vector Model, the predicted OI (OI_pre._) of each odor sample was also obtained. Then, the predictive performance of the modified Vector Model was identified through odor intensity matching tests between OI_mea._ and OI_pre._.

### 2.4. Olfactory and Chemical Analysis

The odor threshold (C_Thr._) of each odorant was determined by employing a dynamic olfactometer (AC’SCENT, St. Croix Sensory, Inc., Stillwater, MN, USA). An odorant (chemical concentration = C_i_) was delivered by the dynamic olfactometer in an ascending dilution series, and the sensory panel measured the averaged dilution multiple (*n*) when the odorant became odorless. Then, odor threshold was calculated as: C_Thr._ = C_i_/*n*. The OI of an odor sample was rated by referencing to the odor intensity referencing scale (OIRS) [12]. Water solutions of 1-butanol were respectively prepared at eight 500 mL Erlenmeyer flasks (room temperature was 25 ± 1 °C) from solution concentration of 12 ppm to 1550 ppm with a geometric progression of two. Assessors compared the olfactory stimulations between odor sample and the OIRS, and then a best match point on the OIRS was determined. For each odor sample, its odor intensity was calculated as the average of all the scores rated by assessors in the sensory panel. The chemical concentrations of odor samples were measured by gas chromatography (GC-2014, Shimadzu, Kyoto, Japan) with a flame ionization detector (GC-FID). The Rtx-5 capillary column (30 m × 0.25 mm ID, 0.5 μm film thickness) was employed and the carrier gas was nitrogen (≥99.999%) at 1.0 mL/min. The injection port of gas chromatography was 200 °C. The column oven temperature was set to 50 °C for 1 min and up to 200 °C at 10 °C·min^−1^ and held for 5 min.

## 3. Results and Discussion

### 3.1. The Linear OI-lnOAV Relation of Individual Odorants

**Figure 2:**
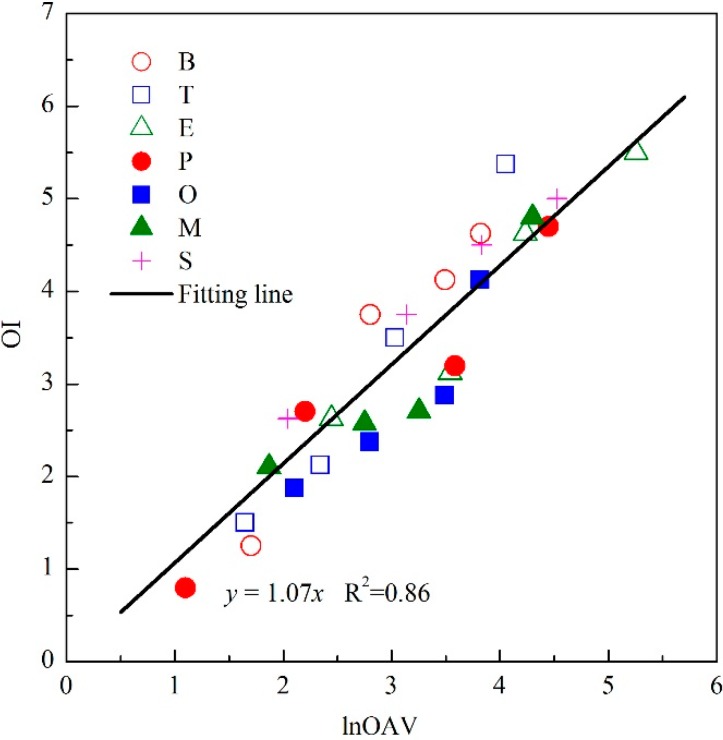
Relationship between odor intensity (OI) and logarithm of odor activity value (lnOAV) of individual aromatic compounds.

The odor threshold (C_Thr._) of each odorant (*i.e.*, B, T, E, P, O, M and S) was firstly measured by the sensory panel, and the results were listed in Table 1. After that, each odorant was prepared in the form of four odor samples with different chemical concentrations. In addition to measuring the OI of each odor sample, its chemical concentration (C_i_) was also measured by gas chromatography. Then, the OAV of each odor sample was calculated (OAV = C_i_/C_Thr._). The OI of each odor sample was rated by nine assessors in the sensory panel, and their averaged value was calculated as the measured OI. As depicted in Figure 2, the results of all seven of these aromatic compounds followed a linear relation between OI and lnOAV. Previous study also had reported the same linear relation of individual benzene, toluene, ethylbenzene and *o*-xylene samples [20]. Besides, Kim *et al.* also reported a similar linear relationship between OI and dilution-to-threshold ratio (D/T ratio, which was similar to the implication of OAV) among individual aldehydes and reduced sulfur compounds [21,22]. According to the literature, the observed linear relation of odorants within a same category was mainly contributed to by their same chemical functional groups and similar molecular structures [23]. The specific fitting equation of tested individual aromatic compounds was OI = 1.07lnOAV (Figure 2). Thus, each lnOAV unit of these aromatic compounds always generated the same strength of olfactory stimulation when it existed individually. Because the olfactory evaluation was easily influenced by many factors including age, gender of assessor and other environmental conditions, the fluctuation of testing results has been widely recognized. If the linear relation was individually performed for each individual odorant, the fitting effect would be better (Figure 2), but it was considered to be a simplification of the Vector Model when all these aromatic compounds were fitted to one line. Thus, the difference of an individual odorant’s fitting precision was neglected. The odor thresholds were usually employed as constants and many related experimental results have been reported. Thus, the OAV of an odorant was easily calculated on the basis of its measured chemical concentration. Based on the obtained linear relation between OI and lnOAV of these aromatic compounds (Figure 2), the OI of an unmixed component in the Vector Model (e.g., OI_a_, OI_b_ in Equation (1)) could be replaced with its corresponding lnOAV value.

### 3.2. The Modification of Vector Model for Binary Odor Mixture

**Figure 3:**
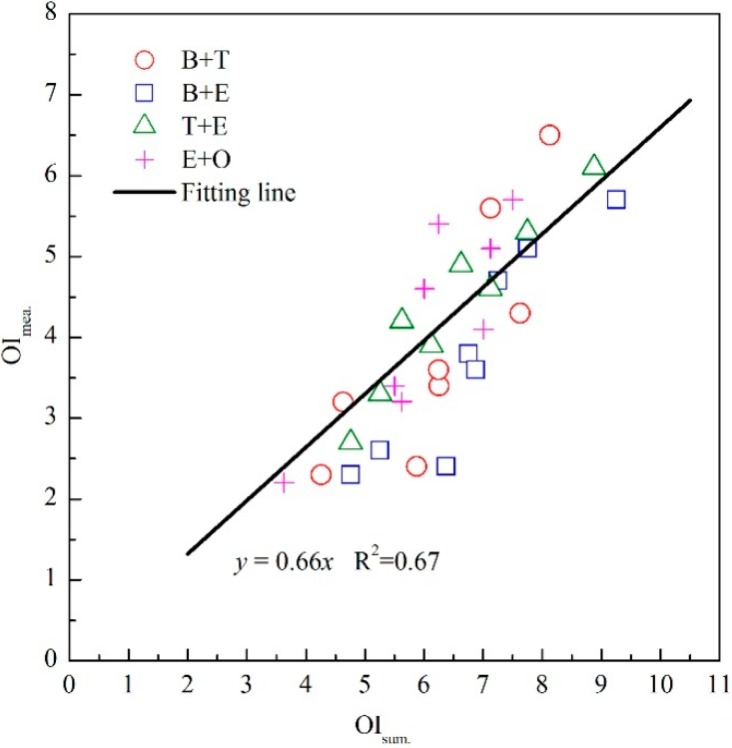
Relationship between OI of a binary odor mixture (OI_mea._) and the summation of its unmixed constituents’ odor intensities (OI_sum._).

Four binary odor mixtures of aromatic compounds (e.g., odor mixtures of B and T; B and E; T and E; E and O) were individually prepared as eight different odor samples. The OI of each odor sample and the odor intensities of its unmixed constituents were all measured through sensory evaluation by the sensory panel. As depicted in Figure 3, the measured OI of a binary odor mixture (OI_mea._) and the summation of its unmixed constituents’ odor intensities (OI_sum._) showed a linear relationship (*i.e.*, OI_mea._ = 0.66OI_sum._). Based on the obtained linear equation, it could be concluded that a distinct counteraction existed in the components’ odor interaction [24]. Furthermore, all these four binary odor mixtures could be fitted into a same linear equation, thus probably indicating a similarity of the odor interactions among these binary odor mixtures.

Because the OI_ab_ in Equation (2) represents the perceived OI of a binary mixture, it could be replaced with the summation of the constituents’ odor intensities (*i.e.*, OI_sum._) on the basis of the obtained linear relation between OI_mea._ and OI_sum._ (Figure 3, OI_mea._ = 0.66OI_sum._ = 0.66(OI_a_ + OI_b_)). According to the determination method of cosα in the Vector Model, the two constituents should be prepared with equal perceived intensities (OI_a_ = OI_b_) [16]. Thus, Equation (2) could be recalculated following Equation (5):

(5) $$\text{cos }{\text{α}}_{\text{ab}}=\frac{{\left(0.66∙\left({\text{OI}}_{a}+{\text{OI}}_{b}\right)\right)}^{2}-{\text{OΙ}}_{a}^{2}-{\text{OΙ}}_{b}^{2}}{2∙{\text{OI}}_{a}∙{\text{OI}}_{b}}=\frac{{\left(0.66\times 2\times {\text{OI}}_{a}\right)}^{2}-2{\text{OΙ}}_{a}^{2}}{2{\text{OΙ}}_{a}^{2}}=\frac{1.74{\text{OΙ}}_{a}^{2}-2{\text{OΙ}}_{a}^{2}}{2{\text{OΙ}}_{a}^{2}}\;=-0.129$$

Because several different binary odor mixtures of aromatic compounds followed the same linear relation (Figure 3), the cosα values were proved to be a constant (Equation (5)). Therefore, fitting all the binary odor mixtures to a same line actually made an important simplification to the determination method of cosα. Then, Equation (1) would be transformed into the following equation:

(6) $${\text{OΙ}}_{\text{ab}}^{2}={\text{OΙ}}_{a}^{2}+{\text{OΙ}}_{b}^{2}-0.258{\text{OΙ}}_{a}∙{\text{OΙ}}_{b}$$

In order to predict the OI of an odor mixture directly by means of instrumental analysis, the linear relation between OI and lnOAV of individual aromatic compounds was also employed. By replacing the OI_a_ and OI_b_ in Equation (6) with their corresponding lnOAV values (Figure 2, OI = 1.07lnOAV), the OI of a binary odor mixture of aromatic compounds was calculated as Equation (7):

(7) $${\text{OI}}_{\text{ab}}=1.07\sqrt{\text{lnOA}{V_{a}}^{2}+\text{lnOA}{V_{b}}^{2}-0.258{\text{lnOAV}}_{a}{\text{lnOAV}}_{b}}$$

The interaction coefficient cosα between any two constituents of a mixture was always calculated following Equation (2). Thus, the cosα values in Equations (3) and (4) were also identified to be the same (*i.e.*, cosα = −0.129) according to the Equation (5), and the Vector Models of ternary and quaternary odor mixtures (Equations (3) and (4)) of aromatic compounds could also be modified the same as the binary mixture one (Equation (5)). The specific calculation equations for ternary and quaternary odor mixtures were are described below:

(8) $$\begin{array}{r} {\text{OI}}_{\text{abc}}=1.07\sqrt{\text{lnOA}{V_{a}}^{2}+\text{lnOA}{V_{b}}^{2}+\text{lnOA}{V_{c}}^{2}-0.258\times {\text{lnOAV}}_{a}{\text{lnOAV}}_{b}}\\ -0.258\times {\text{lnOAV}}_{a}{\text{lnOAV}}_{c}\\ -0.258\times {\text{lnOAV}}_{b}{\text{lnOAV}}_{c} \end{array}$$

(9) $$\begin{array}{r} {\text{OI}}_{\text{abcd}}=1.07\sqrt{\text{lnOA}{V_{a}}^{2}+\text{lnOA}{V_{b}}^{2}+\text{lnOA}{V_{c}}^{2}+\text{lnOA}{V_{d}}^{2}-0.258\times {\text{lnOAV}}_{a}{\text{lnOAV}}_{b}}\\ -0.258\times {\text{lnOAV}}_{a}{\text{lnOAV}}_{c}\\ -0.258\times {\text{lnOAV}}_{a}{\text{lnOAV}}_{d}\\ -0.258\times {\text{lnOAV}}_{b}{\text{lnOAV}}_{c}\\ -0.258\times {\text{lnOAV}}_{b}{\text{lnOAV}}_{d}\\ -0.258\times {\text{lnOAV}}_{c}{\text{lnOAV}}_{d} \end{array}$$

After measuring the constituents’ chemical concentrations by means of instrumental analysis, the OI of an odor mixture would be directly calculated through the modified Vector Models (Equations (7)–(9)).

### 3.3. The Identification of Modified Vector Model in OI Prediction

**Figure 4:**
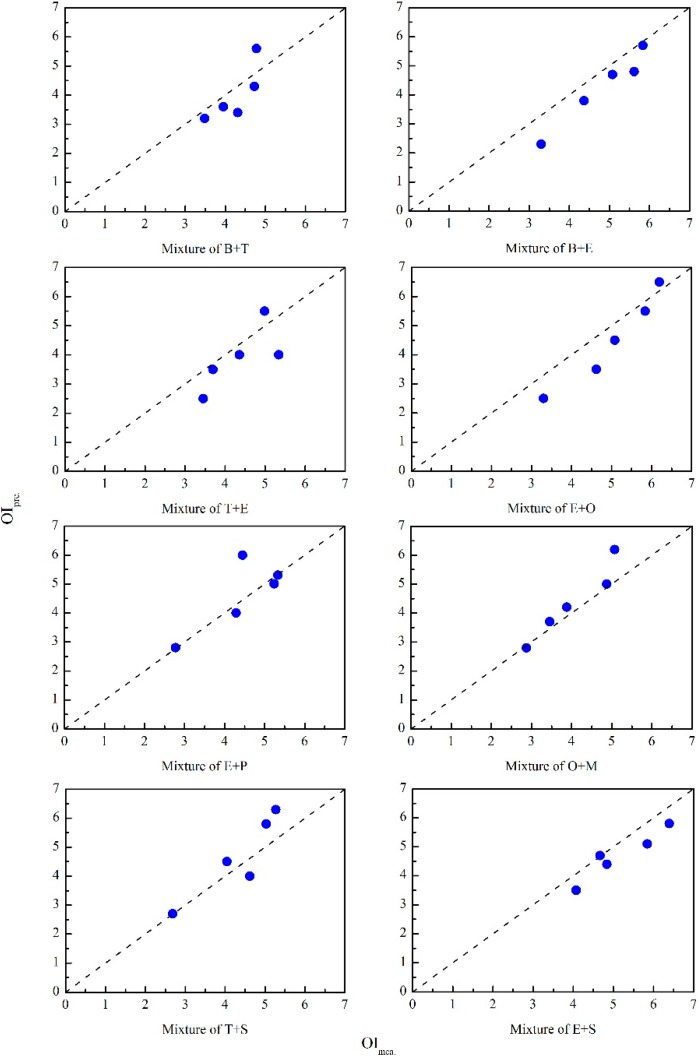
The comparison between measured OI (OI_mea._) and predicted OI (OI_pre._) of eight different binary odor mixtures of aromatic compounds.

As shown in Figure 4, predictive performance of the modified Vector Model was performed by comparison between the measured OI and predicted OI of different odor mixtures. In the above odor intensity matching tests, eight different binary odor mixtures of aromatic compounds were used. Each odor mixture was prepared in the form of five odor samples with various chemical concentration levels. The OI of each odor sample (OI_mea._) was rated by the sensory panel. Besides, the OI of each odor sample (OI_pre._) was also predicted on the basis of its measured chemical concentrations and the modified Vector Model (Equation (5)). The obtained results were depicted by plotting OI_pre._ on the vertical axis and OI_mea._ on the horizontal axis. As shown in Figure 4, the modified Vector Model made predictions that lay close to the diagonal (dashed line) which meant a perfect prediction (OI_mea._ = OI_pre._). In sensory methods utilizing human assessors, fluctuation of the evaluation results in repeated tests (e.g., 0.5 of the OIRS) is normally observed and acknowledged. Thus, random error might be one of the reasons causing the low predictive accuracy of several odor samples in Figure 4.

However, compared with the simple summation of constituents’ odor intensities (Figure 3), the predictive accuracy of modified Vector Model was distinctly better and should contribute to the consideration of odor interactions in the modified Vector Model (*i.e.*, cosα). Although the cosα term was no longer individually calculated for each specific binary odor mixture, the predictive accuracy was still maintained well among all these mixtures. Thus, the simplification of the cosα value (Equation (5)) barely influenced its predictive effect. In contrast, the Vector Model was effectively simplified. After measuring the chemical concentration of an odor mixture’s constituents, its OI could be directly predicted by employing the proposed modified Vector Model and then the evaluation of OI could thus be applied in more fields without the participation of human assessors.

Because odor interactions generally exist between any two constituents of an odor mixture, the degree of odor interaction would be more complex for odor mixtures with greater numbers of components. Thus, the predictive performance of modified Vector Model was also examined for more complex odor mixtures. As listed in Table 2, odor samples of binary, ternary and quaternary odor mixtures were prepared to test the predictive performance of the modified Vector Model. The OI of each odor sample was rated (*i.e.*, OI_mea._) by the sensory panel. Besides, the lnOAV values of each odor sample’s components were also calculated. Based on these lnOAV values, the OI of each odor sample was predicted (*i.e.*, OI_pre._) according to the modified Vector Model (Equations (7)–(9)).

**Table 2:** Comparison of odor intensity between measured values (OI_mea._) and corresponding predicted values (OI_pre._).

Odor Mixture	Concentration of Each Component				OI_mea._	OI_pre._	
	lnOAV_a_	lnOAV_b_	lnOAV_c_	lnOAV_d_		MVM^I^	SCM^II^
T (a) + E (b)	3.36	3.54	-	-	5.4	4.9	3.8
	3.03	2.44	-	-	4.0	3.9	3.2
	2.67	2.44	-	-	3.3	3.6	2.9
T (a) + S (b)	1.65	2.04	-	-	2.7	2.6	2.2
	3.36	2.04	-	-	4.5	4.0	3.6
	2.34	4.53	-	-	6.2	5.2	4.8
T (a) + E (b) + M (c)	2.34	3.54	2.80	-	3.8	4.7	3.8
	2.34	2.44	3.49	-	5.0	4.5	3.7
	1.65	1.78	2.15	-	3.5	3.0	2.3
E (a) + P (b) + S (c)	3.54	2.20	3.83	-	5.4	5.3	4.1
	4.24	2.20	4.53	-	6.8	6.2	4.8
	2.82	2.20	2.65	-	4.5	4.1	3.0
T (a) + E (b) + M (c) + S (d)	4.24	2.20	3.83	2.15	4.8	5.5	4.5
	2.20	3.15	2.20	2.15	4.5	4.1	3.4
	1.85	1.77	2.20	2.15	3.5	3.3	2.4
Average of OI_pre._/OI_mea._						0.96	0.78

^I^MVM: modified Vector Model; ^II^ SCM: Strongest Component Model.

Besides, the strongest component model (SCM) was also employed to compare with the modified Vector Model. The SCM usually calculates the OI of an odor mixture as the biggest value of its unmixed components’ odor intensities. In this study, the component with a higher lnOAV value was firstly selected for each odor sample. Then, the OI of an odor sample (OI_pre._) was calculated on the basis of the selected lnOAV and the fitting formula in Figure 2 (OI = 1.07lnOAV). For instance, the biggest lnOAV value of a ternary mixture E + P + S (e.g., lnOAV of E, P and S were respectively 3.54, 2.20 and 3.83) was 3.83. By employing the SCM, the OI_pre._ of this odor sample was calculated as: OI_pre._ = 1.07 × 3.83 = 4.1. In order to compare the predictive performance between the SCM and modified Vector Model, the average of all the samples’ predictive coefficients (ratio of OI_pre._ and OI_mea._) was calculated for each model, respectively. When the predictive coefficient was 1.0, the predicted OI and measured OI were considered to be the same. As shown in Table 2, the averaged predictive coefficient was 0.96 for the modified Vector Model and 0.78 for the SCM, thus proving that the modified Vector Model was effective for complex odor mixtures and its predictive accuracy was distinctly better than that of the SCM.

In this study, the interaction of odorants was observed to be similar among odorants with the same functional groups and similar molecular structures (Figure 3). Actually, this phenomenon was also observed in other categories of odorants [20,25]. Based on that, the observed linear OI-lnOAV relation (Figure 2) and linear OI_mea._-OI_sum._ relation (Figure 3) also possibly exist among other categories of odorants. Even if both the fitting formula of OI-lnOAV relation and the cosα value were different from this study, the Vector Model modification method is still applicable for other odorants. Usually, olfactory evaluation is carried out together with some form of instrumental analysis, and it provides a more intuitive description to the degree of air pollution. Although sometimes the predictive results of OI were not close enough to the real OI perceived by a human assessor, it was still valuable as a reference for the instrumental analysis. Thus, the modified Vector Model could provide a convenient and more feasible method for sensory evaluation in general indoor and outdoor air pollution assessments.

## 4. Conclusions

In this paper, a modified Vector Model for odor mixtures of aromatic compounds was proposed. Based on the similarity between the measured OI and the summation of constituents’ odor intensities between binary odor mixtures, the determination method of Vector Model’s interaction constant (cosα) was effectively simplified. Besides, the OI of mixture’s unmixed constituents were also replaced by their corresponding lnOAV values on the basis of the linear relation between OI and lnOAV of individual aromatic compounds. Then, the OI of an odor mixture was successfully related with the lnOAV values of its components. For other compounds with similar molecular structures and functional groups, similar modified Vector models produced by employing the methods proposed in this study are also feasible. After a series of odor intensity matching tests for binary, ternary and quaternary odor mixtures, the predictive performance and feasibility of the modified Vector Model were identified to be good. In certain indoor/outdoor air pollution environments, the chemical concentrations of each pollutant could be easily measured by instrumental analysis. Then, the OI of an odor mixture could be directly calculated by employing the corresponding modified Vector Model. Besides the quantitative analysis for targeted compounds, the predicted OI also provided valuable reference for the evaluation of air pollution, and then the modified Vector Model was thus considered helpful in normal air quality assessments.
